# Hydrogen Peroxide Alleviates Nickel-Inhibited Photosynthetic Responses through Increase in Use-Efficiency of Nitrogen and Sulfur, and Glutathione Production in Mustard

**DOI:** 10.3389/fpls.2016.00044

**Published:** 2016-02-03

**Authors:** M. I. R. Khan, Nafees A. Khan, Asim Masood, Tasir S. Per, Mohd Asgher

**Affiliations:** Department of Botany, Aligarh Muslim UniversityAligarh, India

**Keywords:** glutathione, hydrogen peroxide, nickel, photosynthesis, photosynthetic-NUE, photosynthetic-SUE

## Abstract

The response of two mustard (*Brassica juncea* L.) cultivars differing in photosynthetic capacity to different concentrations of hydrogen peroxide (H_2_O_2_) or nickel (Ni) was evaluated. Further, the effect of H_2_O_2_ on photosynthetic responses of the mustard cultivars grown with or without Ni stress was studied. Application of 50 μM H_2_O_2_ increased photosynthesis and growth more prominently in high photosynthetic capacity cultivar (Varuna) than low photosynthetic capacity cultivar (RH30) grown without Ni stress. The H_2_O_2_ application also resulted in alleviation of photosynthetic inhibition induced by 200 mg Ni kg^-1^ soil through increased photosynthetic nitrogen-use efficiency (NUE), sulfur-use efficiency (SUE), and glutathione (GSH) reduced production together with decreased lipid peroxidation and electrolyte leakage in both the cultivars. However, the effect of H_2_O_2_ was more pronounced in Varuna than RH30. The greater increase in photosynthetic-NUE and SUE and GSH production with H_2_O_2_ in Varuna resulted from higher increase in activity of nitrogen (N) and sulfur (S) assimilation enzymes, nitrate reductase and ATP-sulfurylase, respectively resulting in enhanced N and S assimilation. The increased N and S content contributed to the higher activity of ribulose-1,5-bisphosphate carboxylase under Ni stress. Application of H_2_O_2_ also regulated PS II activity and stomatal movement under Ni stress for maintaining higher photosynthetic potential in Varuna. Thus, H_2_O_2_ may be considered as a potential signaling molecule for augmenting photosynthetic potential of mustard plants under optimal and Ni stress conditions. It alleviates Ni stress through the regulation of stomatal and non-stomotal limitations, and photosynthetic-NUE and -SUE and GSH production.

## Introduction

Nickel (Ni) is an essential metallic micronutrient with important roles in plant metabolism. However, excess Ni added to soil due to the increased anthropogenic activities becomes toxic and affects growth and development of plants adversely (Chen et al., 2009; Anjum et al., 2014). The inhibition of metabolic processes in plants by high Ni is associated with the production of excess reactive oxygen species (ROS) causing injury to DNA, oxidation of proteins and lipids and damage to photosynthetic apparatus (Gajewska et al., 2006; Asgher et al., 2015). It has been shown that hydrogen peroxide (H_2_O_2_) plays dual roles in plants under optimal and stressful environments, as it initiates programmed cell death at high concentration and promotes physiological processes at lower concentration (Dat et al., 2000). It acts as a signaling molecule for acclimation and tolerance to various stress factors such as salt (Li et al., 2011), heat (Bhattacharjee, 2013), cold (İşeri et al., 2013), osmotic (Pongprayoon et al., 2013) chilling (Wang et al., 2010), and cadmium (Zelinová et al., 2013). Thus, studies to establish the role of H_2_O_2_ as a secondary messenger molecule (Neill et al., 2002; Hung et al., 2005) and modulator of abiotic stress responses (Uchida et al., 2002; de Azevedo Neto et al., 2005; Wang et al., 2010; Li et al., 2011; Pongprayoon et al., 2013) in plants under normal or stress conditions have gained attention in recent years. Recently, Ashfaque et al. (2014) have shown that exogenous application of H_2_O_2_ promoted photosynthesis and growth of wheat plants grown under salt stress.

Plants up-regulate production of enzymatic and non-enzymatic antioxidants under stress conditions that cooperatively manage to remove/neutralize or scavenge ROS (Noctor et al., 2012) and maintain high rates of photosynthesis (Foyer and Shigeoka, 2011). The regulation of glutathione (GSH) reduced production through modulating nitrogen (N) and sulfur (S) may help in the alleviation of Ni stress-accrued photosynthetic anomalies in crop plants as the availability of N and S influences GSH production and photosynthesis (Iqbal et al., 2011). Therefore, the increased photosynthetic nitrogen use efficiency (NUE) and photosynthetic sulfur use efficiency (SUE) are expected to alleviate Ni-inhibited photosynthetic responses by maintaining the GSH production. The reduction in photosynthetic-NUE and photosynthetic-SUE has been shown to reduce photosynthesis (Iqbal et al., 2012; Khan and Khan, 2014). There are reports that show increase in photosynthesis with H_2_O_2_ (Ashfaque et al., 2014), but detailed studies on the physiological processes and biochemical mechanisms to alleviate Ni stress are not available. In the present study, we analyzed the effects of exogenous H_2_O_2_ on N and S-assimilation and GSH production to examine whether these processes result in improved photosynthetic responses in two cultivars of mustard that differ in photosynthetic capacity under Ni stress.

## Materials and Methods

### Plant Materials and Growth Condition

Seeds of mustard (*Brassica juncea* L. Czern and Coss.) cultivars, Varuna (high photosynthetic capacity) and RH30 (low photosynthetic capacity; Mobin and Khan, 2007) were surface sterilized with 0.01 g L^-1^ HgCl_2_ solution followed by repeated washings and sown in 23-cm diameter pots filled with reconstituted soil [peat and compost, 4:1 (v/v); mixed with sand, 3:1 (v/v)]. After seedlings establishment, three healthy plants of nearly equal size in each pot were maintained. The pots were kept in the naturally illuminated net house of the Botany Department, Aligarh Muslim University, Aligarh (India) with day/night temperatures at 24°C/18°C (±3°C), relative humidity of 68 ± 5%; and the pots were watered daily with 250 mL deionized water. Experiments were conducted independently to study the effect of 0, 50, 100, and 200 mg Ni kg^-1^ soil or 0, 25, 50, and 100 μM H_2_O_2_ applied basally to plants 15 days after seed germination on lipid peroxidation, GSH content, and photosynthesis and plant dry mass of Varuna and RH30. As 50 μM H_2_O_2_ maximally increased photosynthesis and 200 mg Ni kg^-1^ soil was the most toxic, these concentrations were taken for further study. In another experiment, Varuna and RH30 plants were grown in the similar conditions and were treated with 0 (control) or 50 μM H_2_O_2_ in presence or absence of 200 mg Ni kg^-1^ soil at 15 days after seed germination. Nickel chloride (NiCl_2_) was used for Ni treatment. The experiment followed a factorial randomized complete block design and the number of replicates for each treatment was four (*n* = 4). Sampling of plants was done at 30 days after sowing (DAS) to record different parameters.

### Determination of Lipid Peroxidation and Electrolyte Leakage

Lipid peroxidation in leaves was determined by estimating the content of thiobarbituric acid reactive substances (TBARSs) as described by Dhindsa et al. (1981). Fresh leaf tissues (500 mg) were ground in 0.25% 2-thiobarbituric acid (TBA) in 10% trichloroacetic acid (TCA) using mortar and pestle. After heating at 95°C for 30 min, the mixture was rapidly cooled on ice bath and centrifuged at 10,000 *g* for 10 min. To 1 mL of the supernatant 4 mL 20% TCA containing 0.5% TBA was added. The absorbance of the supernatant was read at 532 nm and corrected for non-specific turbidity by subtracting the absorbance of the same at 600 nm. The content of TBARS was calculated using the extinction coefficient (155 mM^-1^ cm^-1^).

For measuring electrolyte leakage, samples were thoroughly washed with sterile water, weighed and then kept in closed vials with 10 mL of deionized water and were incubated at 25°C for 6 h using shaker and electrical conductivity (EC) was determined (C_1_). Samples were then again kept at 90°C for 2 h and EC was recorded after attaining equilibrium at 25°C (C_2_).

### Assay of Antioxidant Enzymes

Fresh leaf tissue (200 mg) was homogenized with an extraction buffer containing 0.05% (v/v) Triton X-100 and 1% (w/v) polyvinylpyrrolidone (PVP) in potassium phosphate buffer (100 mM, pH 7.0) using chilled mortar and pestle. The homogenate was centrifuged at 15,000 × *g* for 20 min at 4°C. The supernatant obtained after centrifugation was used for the assay of glutathione reductase (GR; EC 1.6.4.2) enzyme. For the assay of ascorbate peroxidase (APX; EC 1.11.1.11), extraction buffer was supplemented with 2 mM ascorbate.

Ascorbate peroxidase activity was determined by the method of Nakano and Asada (1981). Ascorbate peroxidase activity was determined by the decrease in the absorbance of ascorbate at 290 nm due to its enzymatic breakdown. The volume of 1 ml of 50 mM K-phosphate buffer (pH 7.2) contained 0.5 mM ascorbate, 0.1 mM H_2_O_2_, 0.1 mM EDTA, and 0.1 ml enzyme extract. The reaction was allowed to run for 5 min at 25°C. Ascorbate peroxidase activity was calculated by using the extinction coefficient 2.8 mM^-1^ cm^-1^. One Unit of enzyme activity is defined as the amount necessary to decompose 1 μmol of substrate consumed per min at 25°C.

Glutathione reductase activity was determined by the method of Foyer and Halliwell (1976). Glutathione reductase activity was determined by monitoring the glutathione dependent oxidation of NADPH at 340 nm. The assay mixture (3 mL) contained phosphate buffer (25 mM, pH 7.8), 0.5 mM oxidized GSH, 0.2 mM NADPH and the enzyme extract. The activity of GR was calculated by using extinction coefficient at 6.2 mM^-1^ cm^-1^. One unit of enzyme was the amount necessary to decompose 1 μmol of NADPH min^-1^ at 25°C.

Protein content was determined following the method of Bradford (1976) using bovine serum albumin (BSA) as a standard.

### Determination of Content of Reduced Glutathione, Oxidized Glutathione, and Redox State

Glutathione was assayed by an enzymic recycling procedure of Anderson (1985). For specific assay of GSSG, the GSH was masked by derivatization with 2-vinylpyridine. Fresh leaves (500 mg) were homogenized in 2.0 mL of 5% sulphosalicylic acid under cold conditions. The homogenate was centrifuged at 10 000 × *g* for 10 min. A 300 μL aliquot of supernatant was taken and neutralized by addition of 18 μL 7.5 mol L^-1^ triethanolamine. One sample of 150 μL was then used to determine concentration of GSH plus GSSG. Another sample was pretreated with 3 μL 2-vinylpyridine for 60 min at 20°C to mask the GSH by derivatization, to allow the subsequent determination of GSSG alone. In each case, 50 μL aliquots of the two samples were mixed with 700 μL 0.3 mmol L^-1^ NADPH, 100 μL 5,5’ -dithiobis-2-nitrobenzoic acid (DTNB) and 150 μL buffer containing 125 mmol L^-1^ sodium phosphate, 6.3 mmol L^-1^ EDTA (pH 6.5). A 10 μL aliquot of glutathione reductase (5 U mL^-1^) was then added and the change in absorbance at 412 nm monitored at 30°C. Redox state was presented as the ratio of GSH to GSSG.

### Assay of Leaf ATP-Sulfurylase and Sulfur Content

Activity of ATP-S (EC 2.7.7.4) was measured using method of Lappartient and Touraine (1996). Fresh leaf tissues (1.0 g) were ground at 4°C in a buffer consisting of 10 mM Na_2_ EDTA, 20 mM Tris-HCl (pH 8.0), 2 mM dithiothreitol (DTT), and 0.01 g mL^-1^ polyvinylpyrrolidone, using 1:4 (w/v) tissue to buffer ratio. The homogenate was centrifuged at 20,000 *g* for 10 min at 4°C. The supernatant was used for *in vitro* ATP-S assay. The reaction was initiated by adding 0.1 mL of extract to 0.5 mL of the reaction mixture, which contained 7 mM MgCl_2_, 5 mM Na_2_MoO_4_, 2 mM Na_2_ATP, and 0.032 units mL^-1^ of sulfate free inorganic pyrophosphate in 80 mM Tris-HCl buffer (pH 8.0). Another aliquot from the same extract was added to the same reaction mixture but without Na_2_MoO_4_. Incubations were carried out at 37°C for 15 min, after which phosphate was determined using UV-vis spectrophotometer.

Sulfur content was determined in leaf samples digested in a mixture of concentrated HNO_3_ and 60% HClO_4_ (85:1 v/v) using turbidimetric method of Chesnin and Yien (1950).

### Determination of Leaf Nitrate Reductase Activity and Nitrogen Content

Activity of nitrate reductase (NR; EC 1.7.99.4) in leaf was measured by preparing enzyme extract using the method of Kuo et al. (1982). Leaves (1.0 g) were frozen in liquid N_2_, ground to a powder with mortar and pestle and then stored at -80°C. The powder was thawed for 10 min at 4°C and homogenized in a blender with 250 mM Tris-HCl buffer (pH 8.5), containing 10 mM cysteine, 1 mM EDTA, 20 M FAD, 1 mM DTT, and 10% (v/v) glycerol. The homogenate was centrifuged at 10,000 *g* for 30 min at 4°C. Activity of NR was assayed spectrophotometrically as the rate of nitrite production at 28°C adopting the procedure of Nakagawa et al. (1984). The assay mixture contained KNO_3_ (10 mM), HEPES (0.065 M; pH 7.0), NADH (0.5 mM) in phosphate buffer (0.04 mM; pH 7.2) and the enzyme in a final volume of 1.5 mL. The reaction was started by adding 0.5 mL NADH. After 15 min, the reaction was terminated by adding 1 mL of 1 N HCl solution containing 1% sulfanilamide followed by the addition of 1 mL of 0.02% aqueous *N*-1-napthylethylene-di-amine-dihydrochloride. The absorbance was read at 540 nm after 10 min.

Leaf N content was determined in acid-peroxide digested material using the method of Lindner (1944).

### Determination of Photosynthetic-NUE and-SUE

Photosynthetic-NUE and -SUE were calculated by the ratio of net photosynthesis to N and S content per unit leaf area, respectively.

### Measurement of PS II Activity

Fully expanded leaves were allowed to adapt under dark for 30 min before chlorophyll fluorescence measurements using Junior-PAM chlorophyll fluorometer (Heinz Walz, Germany) were made. Minimal fluorescence (Fo) and maximum fluorescence (Fm) were measured in dark-adapted leaves with a low measuring beam at a light intensity of 125 μmol m^-2^ s^-1^, whereas under light-adapted condition, minimal fluorescence (Fo’) and maximum fluorescence (Fm’) were measured in the same leaves with a saturating light intensity (720 μmol m^-2^ s^-1^) together with steady-state fluorescence (Fs). The variable fluorescence (F*v* and F*v*’) was calculated using the values of F_m_–F_o_ and Fm’–Fo’, and actual PSII efficiency (Φ PS II) was determined as Fm’–Fs/Fm’, maximal efficiency of PS II by using F*v*/Fm and intrinsic efficiency of PS II by using F*v*’/Fm’. Using fluorescence parameters determined in both the light- and dark-adapted states, the photochemical quenching (qP) and non-photochemical quenching (NPQ) parameters were calculated. Photochemical quenching was calculated as (Fm’–Fs)/F*v*’ and NPQ as (Fm–Fm’)/Fm’ (Maxwell and Johnson, 2000). Electron transport rate (ETR) was calculated by the following formula: Φ PSII × photosynthetic photon flux density × 0.5 × 0.84 as suggested by Krall and Edwards (1992).

### Measurement of Leaf Gas Exchange Parameters

Gas exchange parameters (net photosynthesis, stomatal conductance, and intercellular CO_2_ concentration) were measured in fully expanded uppermost leaves of plants using infrared gas analyzer (CID-340, Photosynthesis System, Bio-Science, USA). The measurements were done on a sunny day at light saturating intensity; PAR; 720 μmol m^-2^ s^-1^ and at 370 ± 5 μmol mol^-1^ atmospheric CO_2_ concentrations.

### Determination of Rubisco Activity and Chlorophyll Quantification

The activity of ribulose 1,5-bisphosphate carboxylase (Rubisco; EC 4.1.1.39) was determined adopting the method of Usuda (1985) by monitoring NADH oxidation at 30°C at 340 nm. For enzyme extraction, leaf tissue (1.0 g) was homogenized using a chilled mortar and pestle with ice-cold extraction buffer containing 0.25 M Tris-HCl (pH 7.8), 0.05 M MgCl_2_, 0.0025 M EDTA, and 37.5mg DTT. This homogenate was centrifuged at 4°C at 10,000 *g* for 10 min. The resulting supernatant was used to assay the enzyme. The reaction mixture (3 mL) contained 100 mM Tris-HCl (pH 8.0), 40 mM NaHCO_3_, 10 mM MgCl_2_, 0.2 mM NADH, 4 mM ATP, 5 mM DTT, 1 U of glyceraldehyde 3-phosphodehydrogenase, 1 U of 3-phosphoglycerate kinase and 0.2 mM ribulose 1,5-bisphosphate (RuBP).

Chlorophyll was extracted without maceration by cutting the leaves and incubating them in 30 mL of dimethyl sulfoxide (DMSO) and acetone mixture (1:1) in dark at 25°C for 30 min (Hiscox and Israelstam, 1979) and the content was quantified using the method of Arnon (1949).

### Measurement of Growth Characteristics

Plants were uprooted, washed and dried on blotting paper, then were kept in an oven at 80°C till constant weight was obtained. The samples were weighed to obtain dry mass with the help of electronic balance. Leaf area was measured using a leaf area meter (LA211, Systronics, New Delhi, India).

### Statistical Analysis

Data were analyzed statistically using analysis of variance (ANOVA) by SPSS 17.0 for Windows, and presented as treatment mean ± SE (*n =* 4). Least significant difference (LSD) was calculated for the significant data at *P* < 0.05. Data followed by same letter are not significantly different by LSD test at *P* < 0.05.

## Results

### Effect of Ni on Lipid Peroxidation, GSH Content, Net Photosynthesis, and Plant Dry Mass

The plants showed differential response to the applied Ni concentrations. The plants receiving lower Ni dose (50 mg Ni kg^-1^ soil) exhibited lower values of leaf TBARS content, whereas the higher Ni doses (100 and 200 mg Ni kg^-1^ soil) increased TBARS content in both the cultivars. The effect of 50 mg Ni kg^-1^ soil in reducing TBARS content was more pronounced in Varuna than RH30. The application of 50 mg Ni kg^-1^ soil reduced TBARS content by 14 and 10.8% in Varuna and RH30, respectively in comparison to control. In contrast, 100 and 200 mg Ni kg^-1^ soil showed more toxic effects; its application resulted in 61 and 147.6% increased TBARS content in Varuna, and 79.6 and 215% in RH30, respectively compared to control (**Table 1**). The increasing Ni concentrations increased GSH content in both the cultivars, but Varuna showed greater response than RH30 and exhibited highest GSH content with 200 mg Ni kg^-1^ soil compared to control. The application of 50, 100, and 200 mg Ni kg^-1^ soil resulted in increase of GSH content by 5.6, 9.5, and 19.4% in Varuna and 4.3, 8.6, and 16.7% in RH30, respectively compared to control (**Table 1**).

**Table 1:** Thiobarbituric acid reactive substance (TBARS) content, GSH content, net photosynthesis, and plant dry mass of Varuna (high photosynthetic capacity) and RH30 (low photosynthetic capacity) cultivars of mustard (*Brassica juncea* L.) at 30 DAS.

	Ni concentration (mg Ni kg^-1^ soil)							
	**Control**		**50**		**100**		**200**	
	
**Parameters**	**Varuna**	**RH30**	**Varuna**	**RH30**	**Varuna**	**RH30**	**Varuna**	**RH30**

TBARS content	21.4 ± 1.1^g^	26 ± 1.0^e^	18.4 ± 0.9^h^	23.2 ± 1.18^f^	34.5 ± 1.8^d^	46.7 ± 2.4^c^	53 ± 3.0^b^	82 ± 4.0^a^
GSH content	284 ± 9.7^d^	210 ± 7^h^	300 ± 11^c^	219 ± 8.0^g^	311 ± 13.8^b^	228 ± 9^f^	339 ± 15^a^	245 ± 10^e^
Net photosynthesis	15.2 ± 0.7^b^	10.1 ± 0.6^e^	17.3 ± 0.8^a^	12 ± 0.6^d^	12.8 ± 0.7^c^	7.4 ± 0.4^g^	9.4 ± 0.49^f^	4.9 ± 0.3^h^
Plant dry mass	5.1 ± 0.23^b^	4.0 ± 0.2^e^	5.8 ± 0.28^a^	4.4 ± 0.21^d^	4.7 ± 0.23^c^	3.3 ± 0.16^g^	3.6 ± 0.2^f^	2.4 ± 0.17^h^

*Plants were basally treated with 0, 50, 100, and 200 mg Ni kg^-1^ soil at 15 days after seed germination. Data are presented as treatments mean ± SE (*n* = 4). Data followed by same letter are not significantly different by LSD test at *P* < 0.05.*

Application of 50 mg Ni kg^-1^ soil promoted net photosynthesis and plant dry mass whereas 100 and 200 mg Ni kg^-1^ soil significantly reduced in both the cultivars with greater reduction in RH30. Application of 50 mg Ni kg^-1^ soil increased net photosynthesis by 13.8% in Varuna and 18.8% in RH30 compared to control. However, the application of 100 and 200 mg Ni kg^-1^ soil reduced net photosynthesis by 15.8 and 8.2% in Varuna and 26.7 and 51.5% in RH30 compared to control (**Table 1**). Application of 50 mg Ni kg^-1^ soil increased plant dry mass by 13.7% in Varuna and 10% in RH30 compared to control. In contrast, application of 100 and 200 mg Ni kg^-1^ soil decreased plant dry mass by 7.8 and 29% in Varuna and 17.5 and 40% in RH30 compared to control (**Table 1**).

### Effect of H_2_O_2_ on Lipid Peroxidation, GSH Content, Net Photosynthesis, and Plant Dry Mass

Application of 25 and 50 μM H_2_O_2_ reduced lipid peroxidation, while 100 μM H_2_O_2_ increased it. The treatment of 50 μM H_2_O_2_ was most effective in reducing lipid peroxidation in both the cultivars, but more prominently in Varuna. Lipid peroxidation was reduced by 42.9 and 28% in Varuna and RH30, respectively with 50 μM H_2_O_2_ in comparison to control. Application of 100 μM H_2_O_2_ proved toxic to both the cultivars and increased lipid peroxidation by 23.8% in Varuna and 28% in RH30 compared to control (**Table 2**). The increasing concentration of H_2_O_2_ increased GSH content, but the increase was more conspicuous in Varuna than RH30. Application of 25, 50, and 100 μM H_2_O_2_ increased GSH content by 19.6, 36, and 44.1% in Varuna and 9.1, 18.8, and 24.5% in RH30 compared to control (**Table 2**).

**Table 2:** Thiobarbituric acid reactive substance content, GSH content, net photosynthesis, and plant dry mass of Varuna (high photosynthetic capacity) and RH30 (low photosynthetic capacity) cultivars of mustard (*Brassica juncea* L.) at 30 DAS.

H_2_O_2_ concentration (μM)								
	**Control**		**25**		**50**		**100**	
**Parameters**	**Varuna**	**RH30**	**Varuna**	**RH30**	**Varuna**	**RH30**	**Varuna**	**RH30**

TBARS content	21 ± 1.1^c^	25 ± 1.0^b^	17 ± 0.85^d^	22 ± 1.1^c^	12 ± 0.68^e^	18 ± 1^d^	26 ± 1.3^b^	32 ± 1.6^a^
GSH content	286 ± 11^d^	208 ± 10^h^	342 ± 16^c^	227 ± 11^g^	389 ± 17^b^	247 ± 12^f^	412 ± 19^a^	259 ± 13^e^
Net photosynthesis	15.4 ± 0.8^c^	10.4 ± 0.7^g^	17.3 ± 0.8^b^	11.3 ± 0.6^f^	19.9 ± 1.1^a^	12.2 ± 0.5^e^	13.1 ± 0.7^d^	7.2 ± 0.4^h^
Plant dry mass	5.2 ± 0.27^c^	3.4 ± 0.21^g^	5.7 ± 0.28^b^	3.8 ± 0.21^f^	6.3 ± 0.34^a^	4.1 ± 0.23^e^	4.5 ± 0.24^d^	2.7 ± 0.16^h^

*Plants were basally treated with 0, 25, 50, and 100 μM H_2_O_2_ mg at 15 days after seed germination. Data are presented as treatments mean ± SE (*n* = 4). Data followed by same letter are not significantly different by LSD test at *P* < 0.05.*

The varied effect of H_2_O_2_ concentration was noted on net photosynthesis and plant dry mass in both the cultivars. Application of 50 μM H_2_O_2_ improved net photosynthesis and plant dry mass more than 25 μM H_2_O_2_ in both the cultivars, with greater increase in Varuna than RH30. The application of 50 μM H_2_O_2_ increased net photosynthesis and plant dry mass by 29.2 and 21.2% in Varuna 17.3 and 20.6% in RH30 compared to control. In contrast, 100 μM H_2_O_2_ decreased net photosynthesis and plant dry mass in both the cultivars, with greater reduction in RH30 (**Table 2**).

### Application of Hydrogen Peroxide Resisted Lipid Peroxidation and Electrolyte Leakage Induced by Ni Stress

**FIGURE 1:**
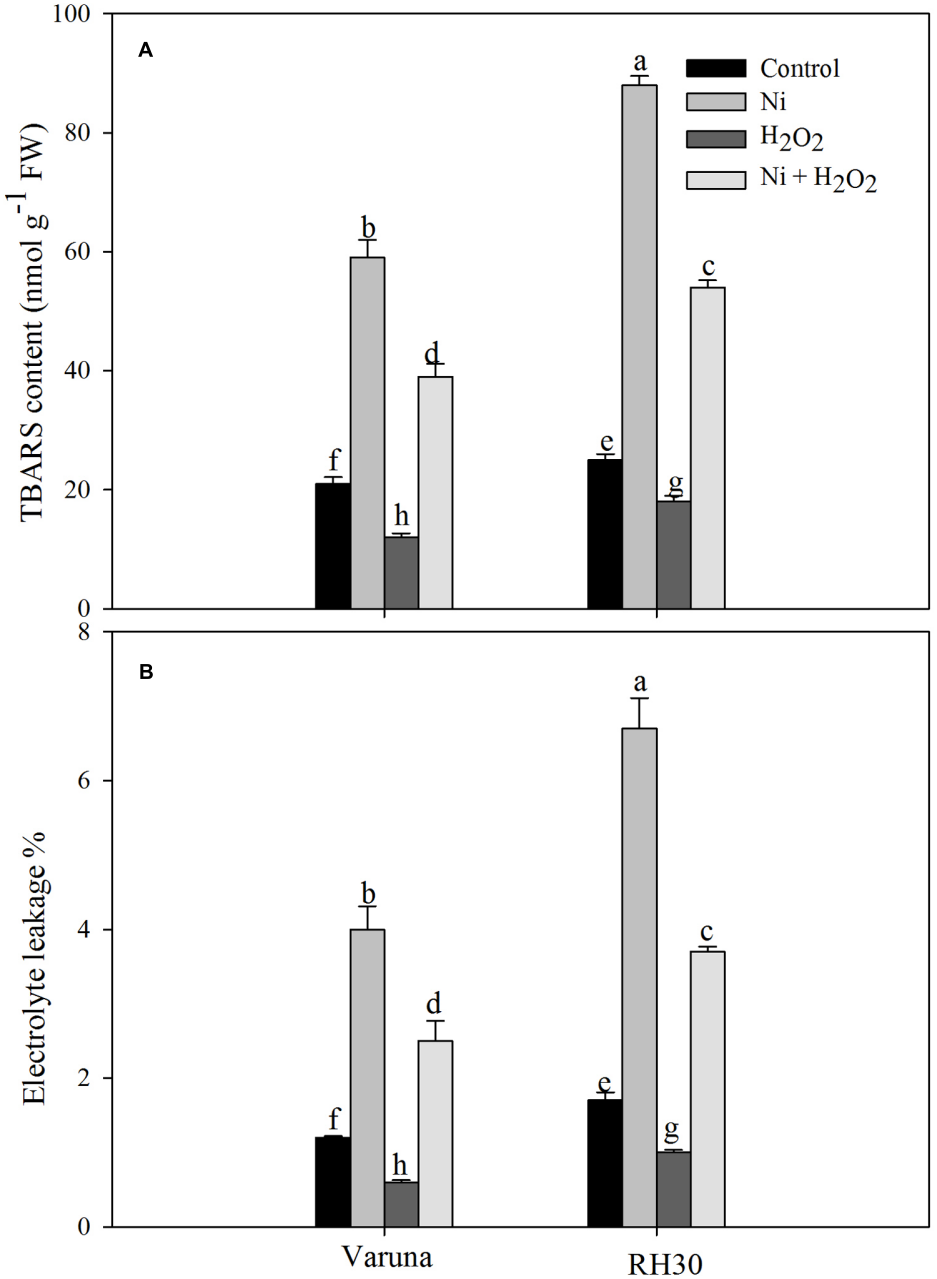
**Thiobarbituric acid reactive substance (TBARS) content **(A)** and electrolyte leakage **(B)** of Varuna (high photosynthetic capacity) and RH30 (low photosynthetic capacity) cultivars of mustard (*Brassica juncea* L.) at 30 DAS.** Plants were basally treated with 0 or 50 μM H_2_O_2_ in presence or absence of 200 mg Ni kg^-1^ soil at 15 days after seed germination. Data are presented as treatments mean ± SE (*n* = 4). Data followed by same letter are not significantly different by LSD test at *P* < 0.05.

Application of Ni increased lipid peroxidation (TBARS content) and electrolyte leakage in both the cultivars, but RH30 exhibited greater increase in TBARS content and electrolyte leakage. The increase in these parameters was 2.8- and 3.3-fold in Varuna, and 3.5- and 3.9-fold in RH30, respectively compared to control plants. Application of hydrogen peroxide reduced lipid peroxidation and electrolyte leakage in both the cultivars but to greater extent in Varuna than RH30 compared to control in no stress condition. Hydrogen peroxide-treated plants showed decrease in TBARS content and electrolyte leakage by 1.8- and 2.0-fold in Varuna, and by 1.3- and 1.6-fold in RH30 compared to control plants. Follow-up treatment of hydrogen peroxide to Ni grown plants resisted the peroxidation and electrolyte leakage resulting in lesser TBARS content and electrolyte leakage equally by 1.5-fold in Varuna and 1.1-fold in RH30 compared to Ni-treated plants (**Figure 1**).

### Hydrogen Peroxide Accelerated Antioxidant System and Maintained Redox Status under Ni Stress

**FIGURE 2:**
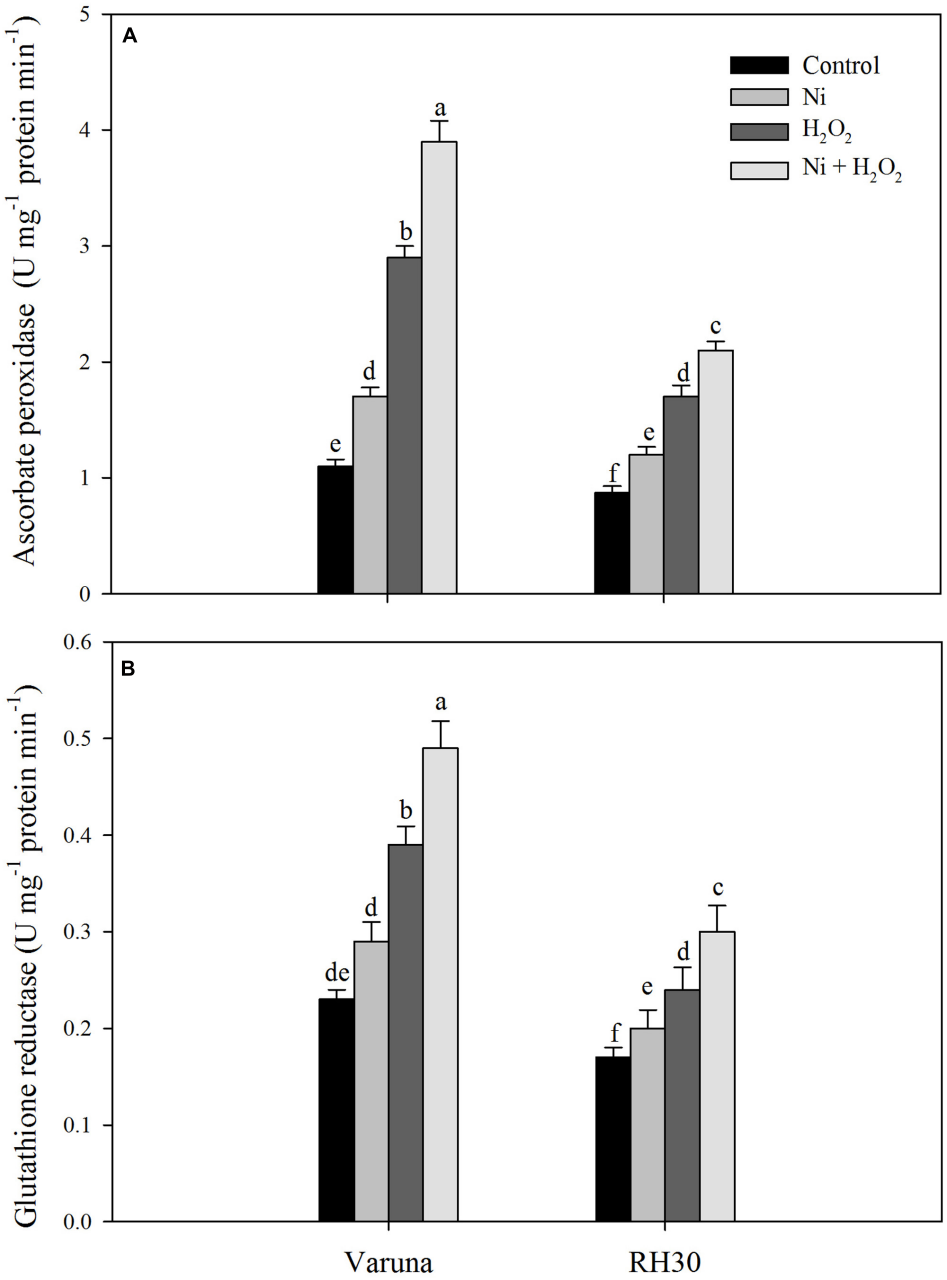
**Activity of ascorbate peroxidase **(A)** and glutathione reductase **(B)** of Varuna (high photosynthetic capacity) and RH30 (low photosynthetic capacity) cultivars of mustard (*Brassica juncea* L.) at 30 DAS.** Plants were basally treated with 0 or 50 μM H_2_O_2_ in presence or absence of 200 mg Ni kg^-1^ soil at 15 days after seed germination. Data are presented as treatments mean ± SE (*n* = 4). Data followed by same letter are not significantly different by LSD test at *P* < 0.05.

The effect of hydrogen peroxide was evaluated on enzymatic and non-enzymatic antioxidants system under Ni stress to assess the involvement of such defense mechanisms in Ni tolerance. The activity of antioxidant enzymes increased to a greater extent in Varuna than RH30. Application of hydrogen peroxide increased activity of APX and GR by 2.6- and 1.8-fold in Varuna and 2.0- and 1.3-fold in RH30 in plants grown without Ni, but by 3.5- and 1.3-fold in Varuna and 2.4- and 1.0-fold in RH30 in plants grown with Ni in comparison to control (**Figure 2**).

**FIGURE 3:**
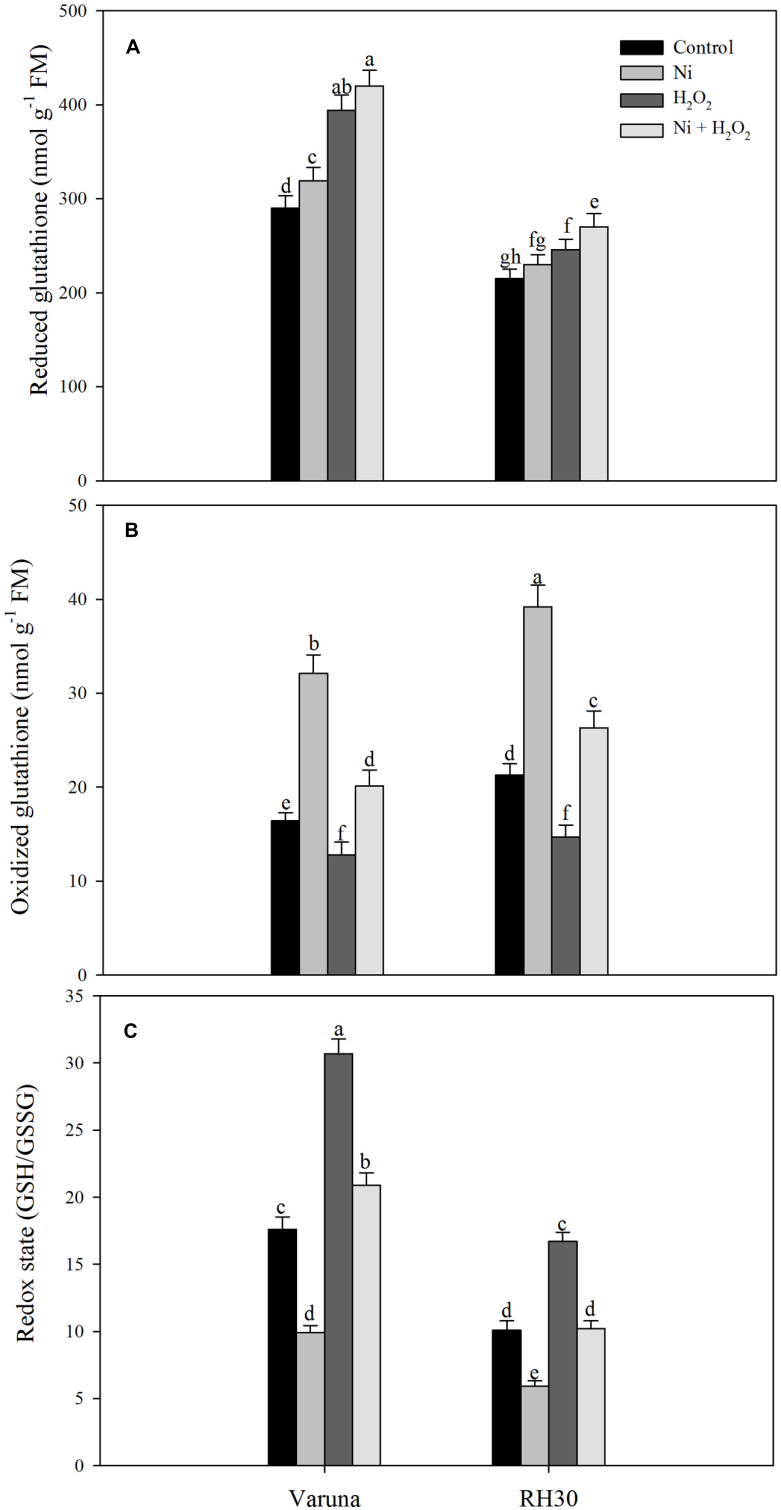
**Content of glutathione (GSH) reduced **(A)**, GSH oxidized (GSSG; **B**) and redox state (GSH/GSSG; **C**) of Varuna (high photosynthetic capacity) and RH30 (low photosynthetic capacity) cultivars of mustard (*Brassica juncea* L.) at 30 DAS.** Plants were basally treated with 0 or 50 μM H_2_O_2_ in presence or absence of 200 mg Ni kg^-1^ soil at 15 days after seed germination. Data are presented as treatments mean ± SE (*n* = 4). Data followed by same letter are not significantly different by LSD test at *P* < 0.05.

Plants grown with Ni showed increased GSH content more prominently in Varuna than RH30. Treatment with hydrogen peroxide in plants grown with Ni further increased the GSH content by 1.5-fold in Varuna and 1.1-fold in RH30 compared to control (**Figure 3**). However, plants grown under Ni stress showed higher GSSG (oxidized glutathione) content more prominently in Varuna than RH30. In contrast, H_2_O_2_-treated plants showed lesser value of GSSG compared to control plants. Redox state of Ni-treated plants reduced compared to control plants, but application of H_2_O_2_ to Ni-treated plants resulted in equal redox state to control plants (**Figure 3**).

### Hydrogen Peroxide Increased N and S Assimilation and Photosynthetic-NUE and -SUE under Ni Stress

**FIGURE 4:**
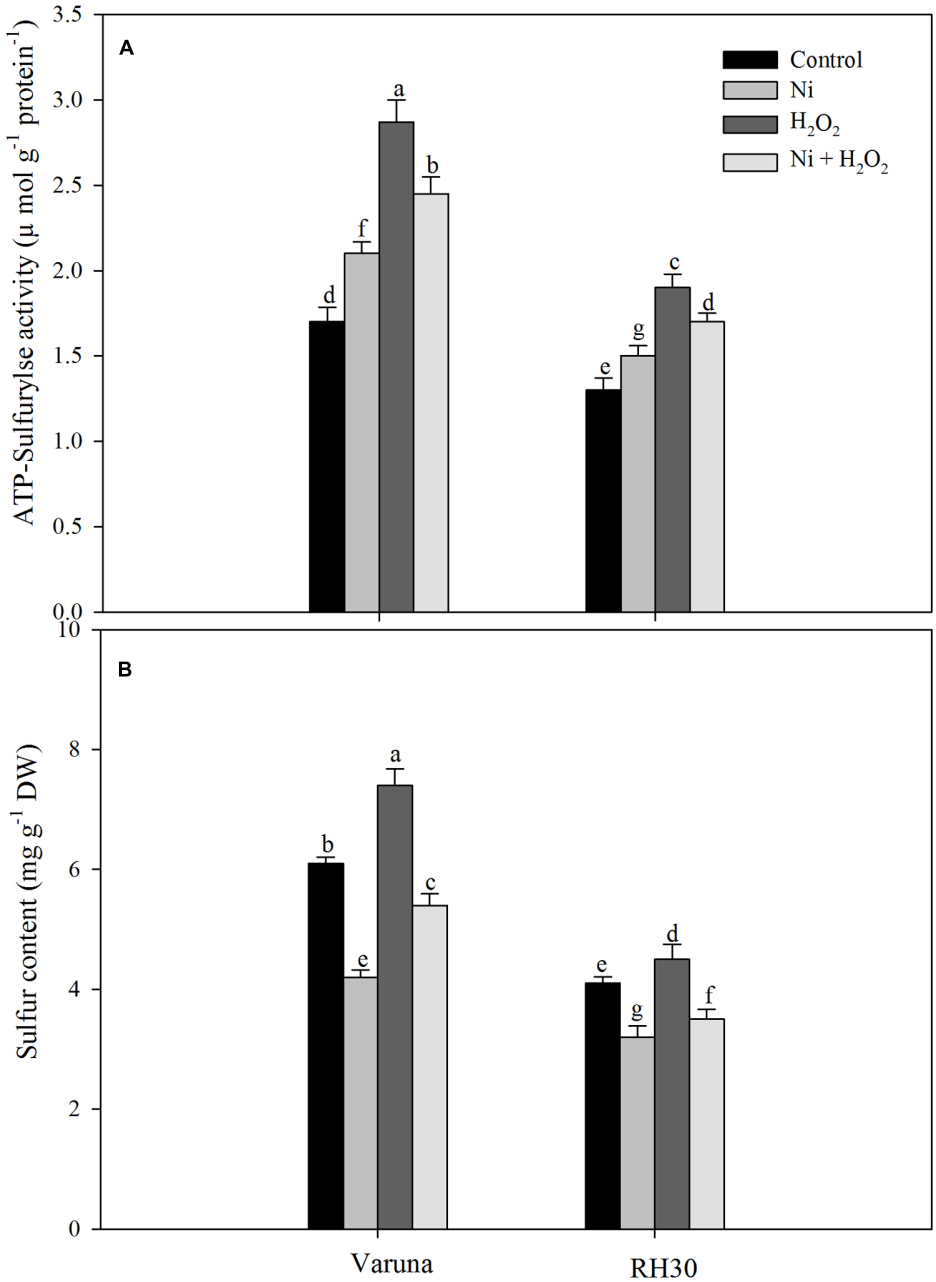
**Leaf ATP-sulfurylse activity **(A)** and sulfur content **(B)** of Varuna (high photosynthetic capacity) and RH30 (low photosynthetic capacity) cultivars of mustard (*Brassica juncea* L.) at 30 DAS.** Plants were basally treated with 0 or 50 μM H_2_O_2_ in presence or absence of 200 mg Ni kg^-1^ soil at 15 days after seed germination. Data are presented as treatments mean ± SE (*n* = 4). Data followed by same letter are not significantly different by LSD test at *P* < 0.05.

Under Ni stress, ATP-S activity increased while S content decreased in both the cultivars compared to control. In the presence of Ni the increase in ATP-S activity was 23.5 and 15.3%, and decrease in S content was 31.1 and 21.9% in Varuna and RH30, respectively compared to control. Application of hydrogen peroxide increased ATP-S activity and S content by 68.8 and 21.3% in Varuna and 46.1 and 9.7% in RH30 compared to control in plants grown without Ni. In the presence of Ni, H_2_O_2_ application also resulted in increase in ATP-S activity and S content in both the cultivars (**Figure 4**).

**FIGURE 5:**
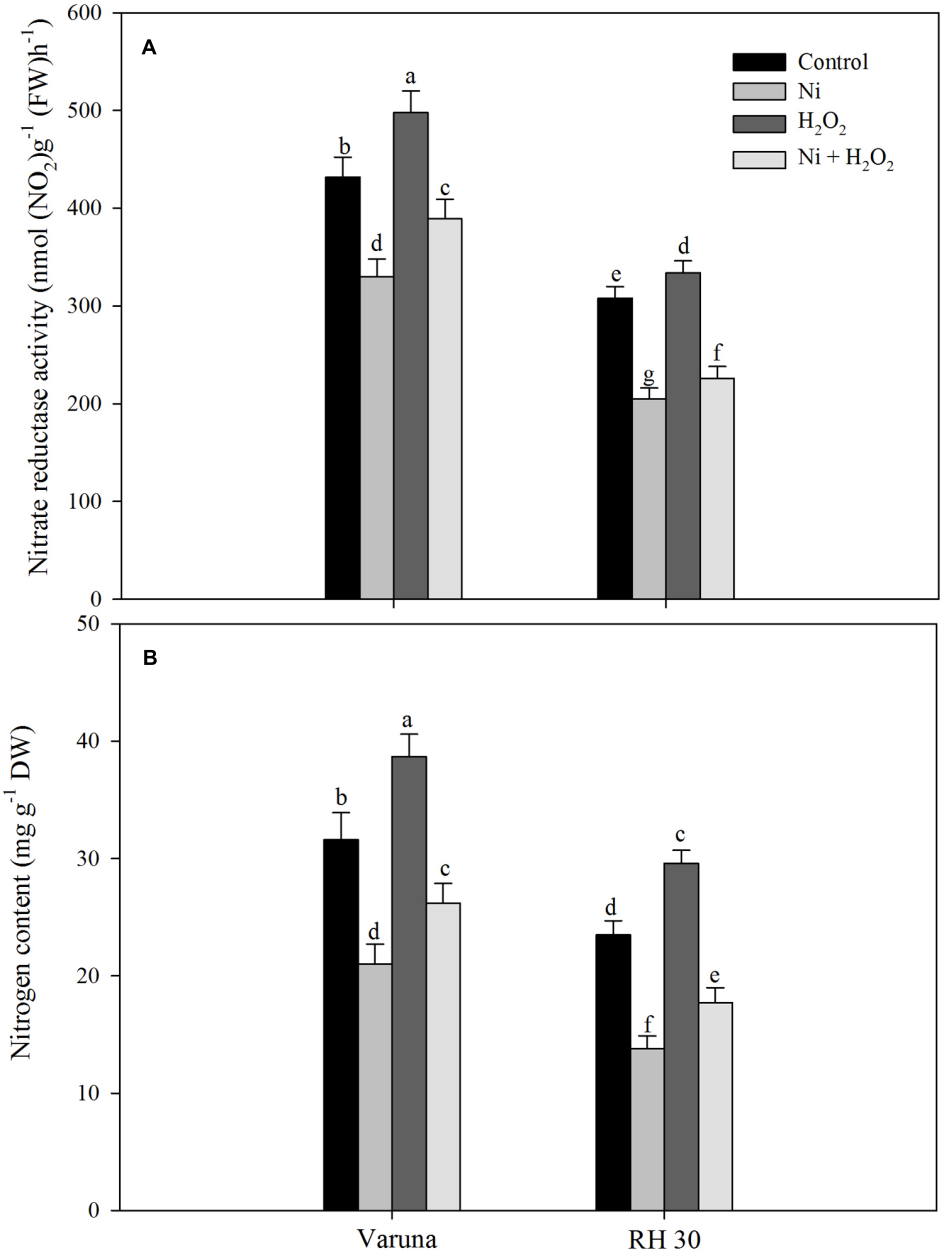
**Leaf nitrate reductase activity **(A)** and nitrogen content **(B)** of Varuna (high photosynthetic capacity) and RH30 (low photosynthetic capacity) cultivars of mustard (*Brassica juncea* L.) at 30 DAS.** Plants were basally treated with 0 or 50 μM H_2_O_2_ in presence or absence of 200 mg Ni kg^-1^ soil at 15 days after seed germination. Data are presented as treatments mean ± SE (*n* = 4). Data followed by same letter are not significantly different by LSD test at *P* < 0.05.

Hydrogen peroxide application influenced N assimilation and S assimilation under no stress and Ni stress conditions. The application of hydrogen peroxide on plants grown with Ni proved beneficial in increasing the activity of NR and N content compared to control plants. Plants treated with Ni showed decrease in NR activity and N content by 23.6 and 33.5% in Varuna, and 33.4 and 41.2% in RH30, respectively compared to control plants. The decrease in NR activity and N content was reduced with hydrogen peroxide by 15.5 and 17.1% in Varuna and 23.1 and 24.6%, respectively in RH30 compared to control (**Figure 5**).

**FIGURE 6:**
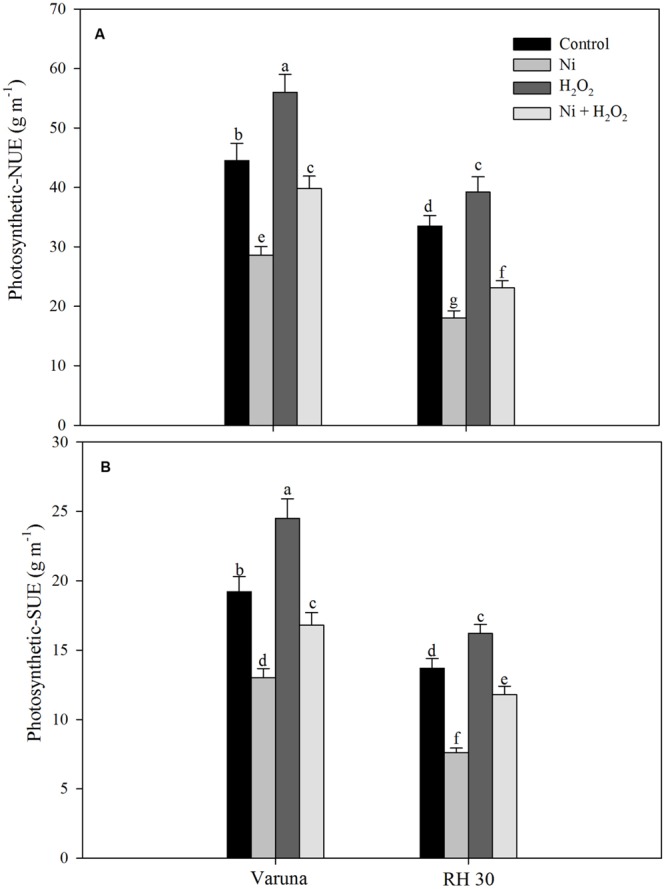
**Photosynthetic-nitrogen use efficiency (NUE; **A**) and photosynthetic-sulfur use efficiency (SUE; **B**) of Varuna (high photosynthetic capacity) and RH30 (low photosynthetic capacity) cultivars of mustard (*Brassica juncea* L.) at 30 DAS.** Plants were basally treated with 0 or 50 μM H_2_O_2_ in presence or absence of 200 mg Ni kg^-1^ soil at 15 days after seed germination. Data are presented as treatments mean ± SE (*n* = 4). Data followed by same letter are not significantly different by LSD test at *P* < 0.05.

Photosynthetic-NUE and-SUE decreased more conspicuously in RH30 with Ni stress compared to control plants. The treatment of Ni decreased photosynthetic-NUE and -SUE by 35.7 and 32.2% in Varuna and 44.5 and 46.2% in RH30 compared to control plants. However, treatment with H_2_O_2_ reduced the toxic effect of Ni and increased photosynthetic-NUE and -SUE by 31.6 and 33.9% in Varuna and 19.0 and 13.9% in RH30, respectively compared to control (**Figure 6**).

### Application of Hydrogen Peroxide Reversed Photosynthetic Inhibition by Ni

**FIGURE 7:**
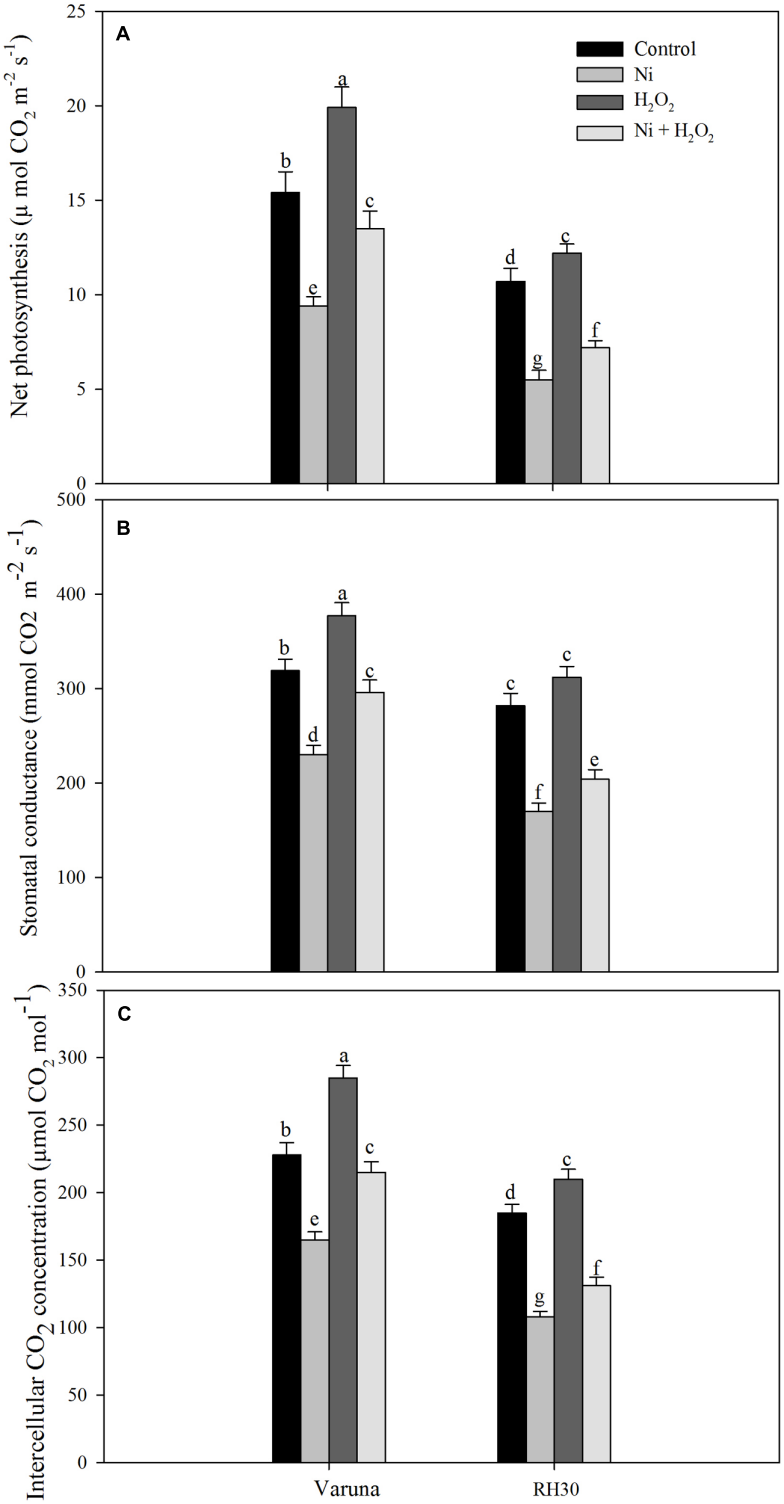
**Net photosynthesis **(A)** stomatal conductance **(B)** and intercellular CO_2_ concentration **(C)** of Varuna (high photosynthetic capacity) and RH30 (low photosynthetic capacity) cultivars of mustard (*Brassica juncea* L.) at 30 DAS.** Plants were basally treated with 0 or 50 μM H_2_O_2_ in presence or absence of 200 mg Ni kg^-1^ soil at 15 days after seed germination. Data are presented as treatments mean ± SE (*n* = 4). Data followed by same letter are not significantly different by LSD test at *P* < 0.05.

Gas exchange parameters were more severely affected in RH30 than Varuna plants under Ni stress. The reduction in net photosynthesis, stomatal conductance, and intercellular CO_2_ concentration with Ni was 38.9, 27.9, and 27.6% in Varuna, while these parameters decreased by 48.6, 39.7, and 41.6%, respectively in RH30 compared to control. Application of hydrogen peroxide increased net photosynthesis, stomatal conductance, and intercellular CO_2_ concentration by 29.2, 18.2, and 25.0% in Varuna and by 14.0, 10.6, and 13.5%, respectively in RH30 in comparison to control. However, application of hydrogen peroxide to Ni-treated plants increased net photosynthesis, stomatal conductance, and intercellular CO_2_ by 43.6, 28.7, and 30.3% in Varuna, and 30.9, 20, and 21.3% in RH30, respectively compared to Ni-treated plants (**Figure 7**).

**FIGURE 8:**
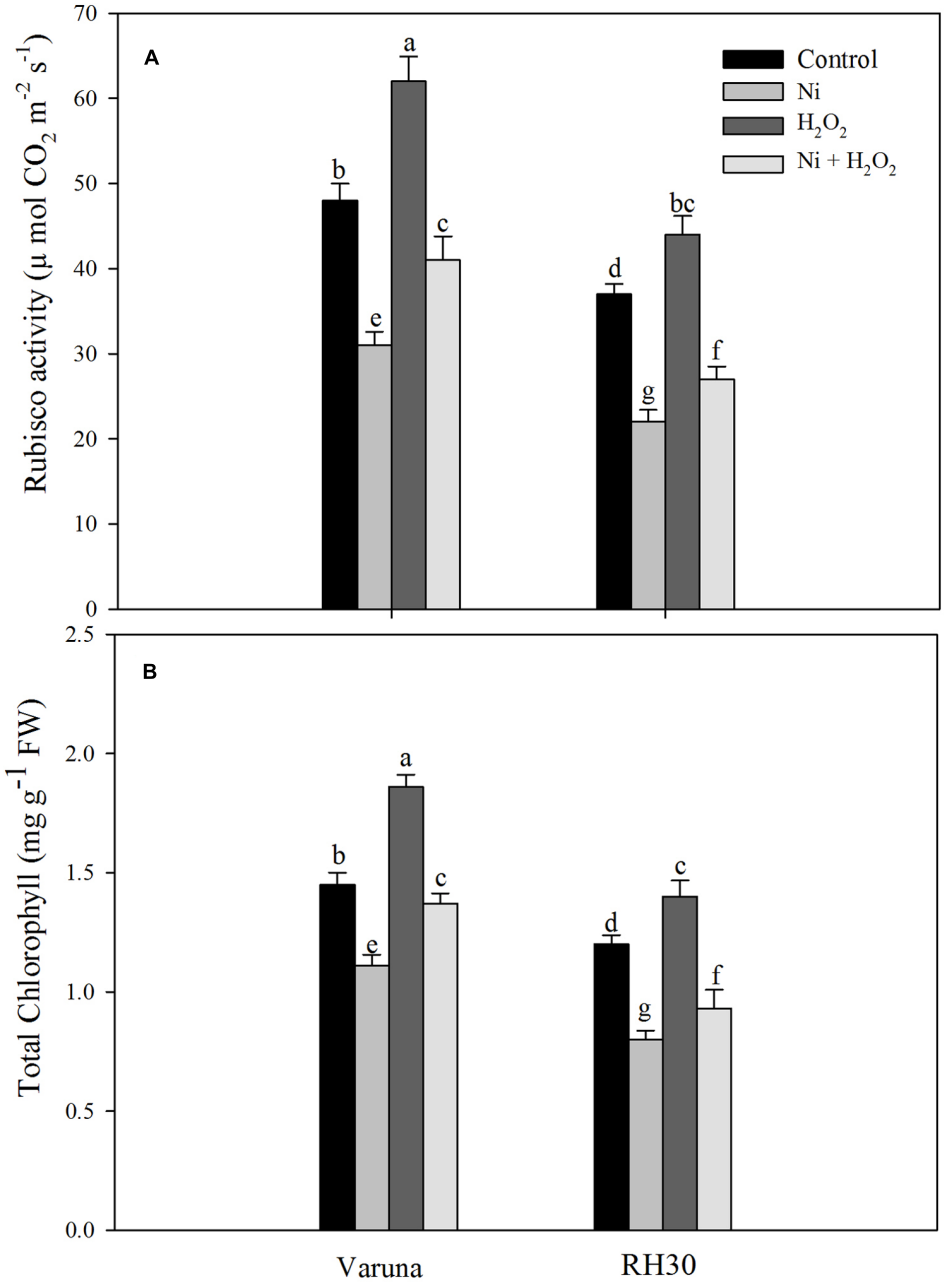
**Rubisco activity **(A)** and chlorophyll content **(B)** of Varuna (high photosynthetic capacity) and RH30 (low photosynthetic capacity) cultivars of mustard (*Brassica juncea* L.) at 30 DAS.** Plants were basally treated with 0 or 50 μM H_2_O_2_ in presence or absence of 200 mg Ni kg^-1^ soil at 15 days after seed germination. Data are presented as treatments mean ± SE (*n* = 4). Data followed by same letter are not significantly different by LSD test at *P* < 0.05.

Rubisco activity decreased more conspicuously in RH30 than Varuna compared to control under Ni treatment. However, hydrogen peroxide reduced the negative effects of Ni by increasing Rubisco activity in both the cultivars by increasing the activity to 32.3% in Varuna and 22.7% in RH30 compared to control. Nickel toxicity also reduced chlorophyll content in both the cultivars more prominently in RH30 compared to control. The chlorophyll content was reduced by 23.4% in Varuna and 33.3% in RH30 with Ni compared to control. The content of chlorophyll increased by 28.3% in Varuna and 16.7% in RH30 in hydrogen peroxide-treated plant compared to control under no stress condition. However, in Ni-treated plants hydrogen peroxide treatment increased chlorophyll content by 23.4 and 16.3% in Varuna and RH30, respectively compared to Ni-treated plants (**Figure 8**).

### Hydrogen Peroxide Modulated PS II Activity under Ni Stress

**FIGURE 9:**
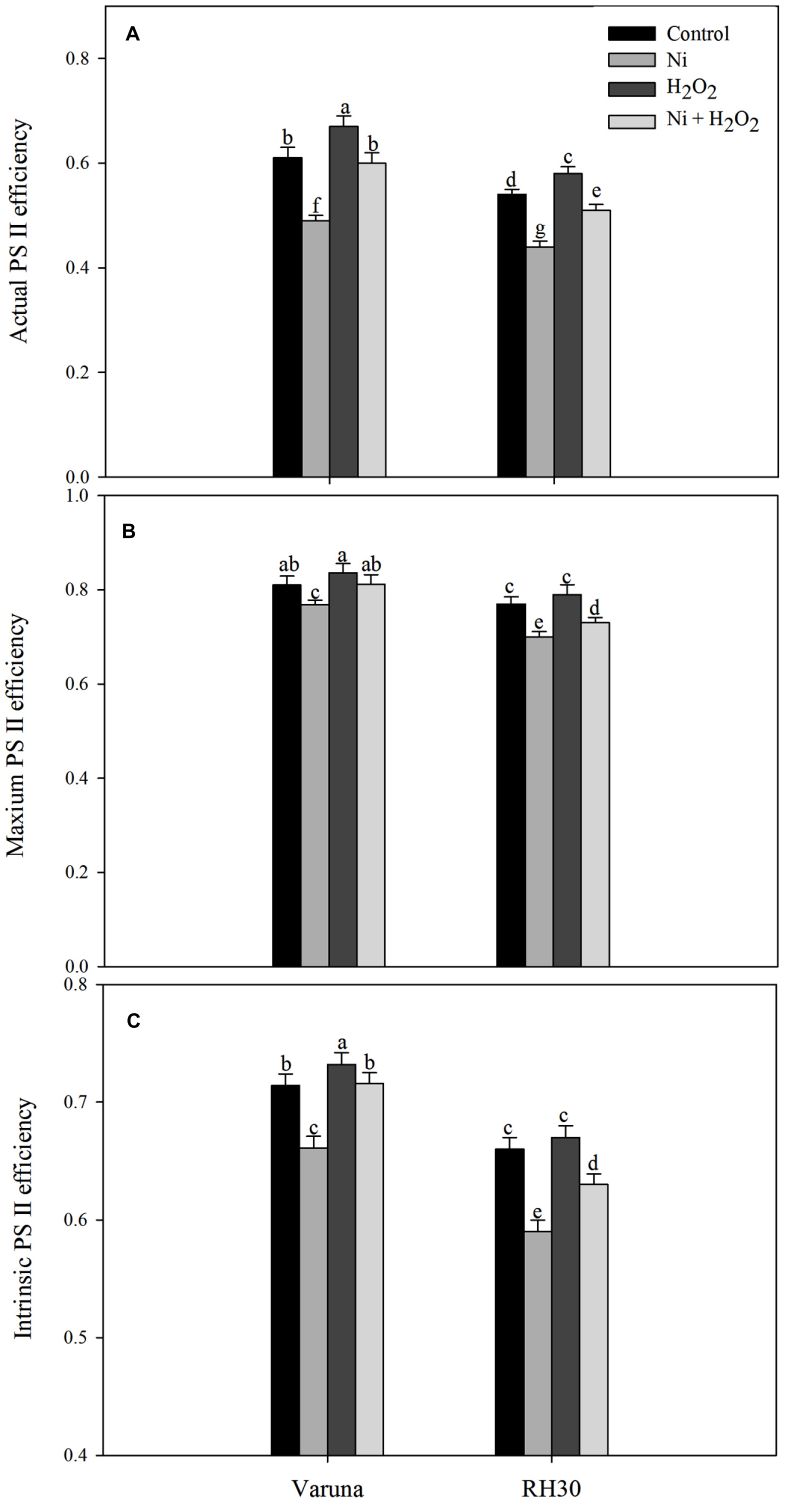
**Actual PS II efficiency **(A)** maximum PS II efficiency **(B)** and intrinsic PS II efficiency **(C)** of Varuna (high photosynthetic capacity) and RH30 (low photosynthetic capacity) cultivars of mustard (*Brassica juncea* L.) at 30 DAS.** Plants were basally treated with 0 or 50 μM H_2_O_2_ in presence or absence of 200 mg Ni kg^-1^ soil at 15 days after seed germination. Data are presented as treatments mean ± SE (*n* = 4). Data followed by same letter are not significantly different by LSD test at *P* < 0.05.

**FIGURE 10:**
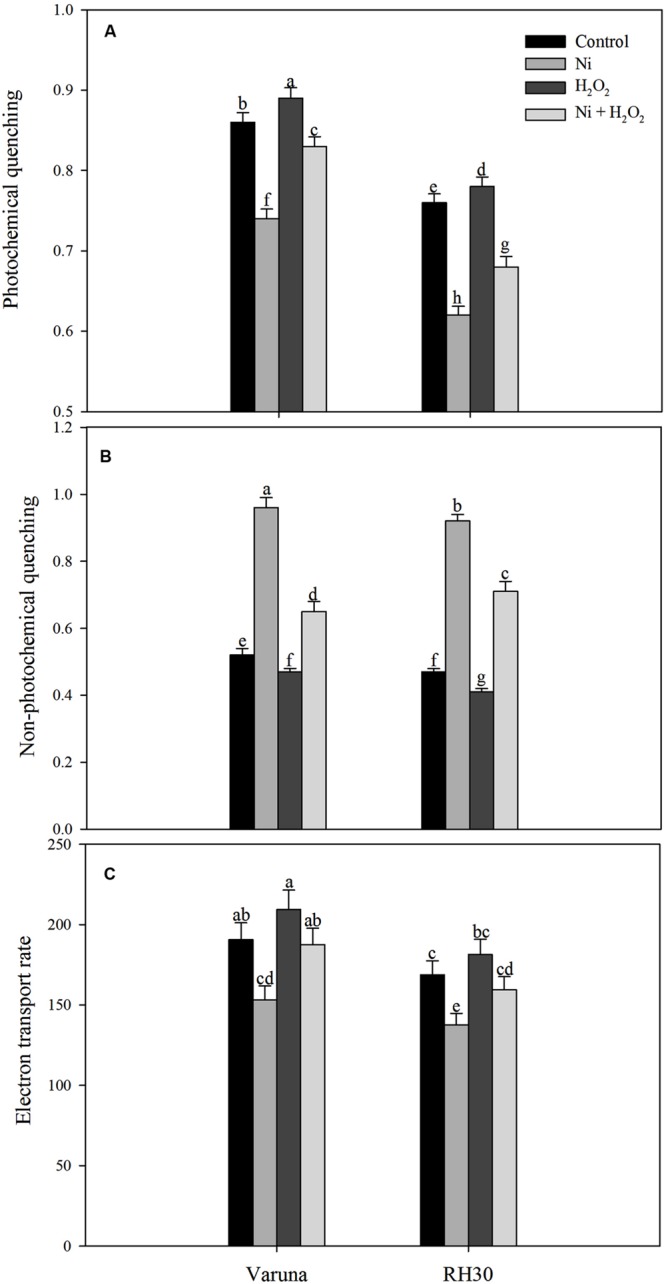
**Photochemical quenching **(A)** non-photochemical quenching **(B)** and electron transport rate **(C)** of Varuna (high photosynthetic capacity) and RH30 (low photosynthetic capacity) cultivars of mustard (*Brassica juncea* L.) at 30 DAS.** Plants were basally treated with 0 or 50 μM H_2_O_2_ in presence or absence of 200 mg Ni kg^-1^ soil at 15 days after seed germination. Data are presented as treatments mean ± SE (*n* = 4). Data followed by same letter are not significantly different by LSD test at *P* < 0.05.

Chlorophyll fluorescence was measured in plants grown with Ni and treated with hydrogen peroxide. Plants grown with Ni exhibited reduced ΦPSII, Fv/Fm, intrinsic efficiency of PSII (Fv’/Fm’; **Figure 9**), ETR, and qP (**Figure 10**) compared to control. These attributes decreased by 19.7, 5.2, 7.4, 19.7, and 14.0% in Varuna and 18.5, 9.1, 10.6, 18.5, and 18.4%, respectively in RH30 in comparison to control plants. However, NPQ increased with Ni treatment by 84.6% in Varuna and by 95.7% in RH30 in comparison to control. Furthermore, NPQ significantly decreased with hydrogen peroxide application in Ni-treated plants in both the cultivars by 32.3 and 22.8%, respectively compared with Ni-treated plants. Follow-up treatment of H_2_O_2_ to Ni-treated plants proved effective in improving ΦPSII along with Fv/Fm, Fv’/Fm’, ETR, and qP compared to Ni stress. Hydrogen peroxide application to plants with Ni stress inhibited the decrease and improved all the above characteristics by 22.4, 5.7, 8.3, 22.5, and 12.2% in Varuna and 16, 4.3, 6.7, 15.9, and 9.6% in RH30, respectively compared with Ni-treated plants (**Figures 9** and **10**).

### Hydrogen Peroxide Protected Growth Characteristics under Ni Stress

**FIGURE 11:**
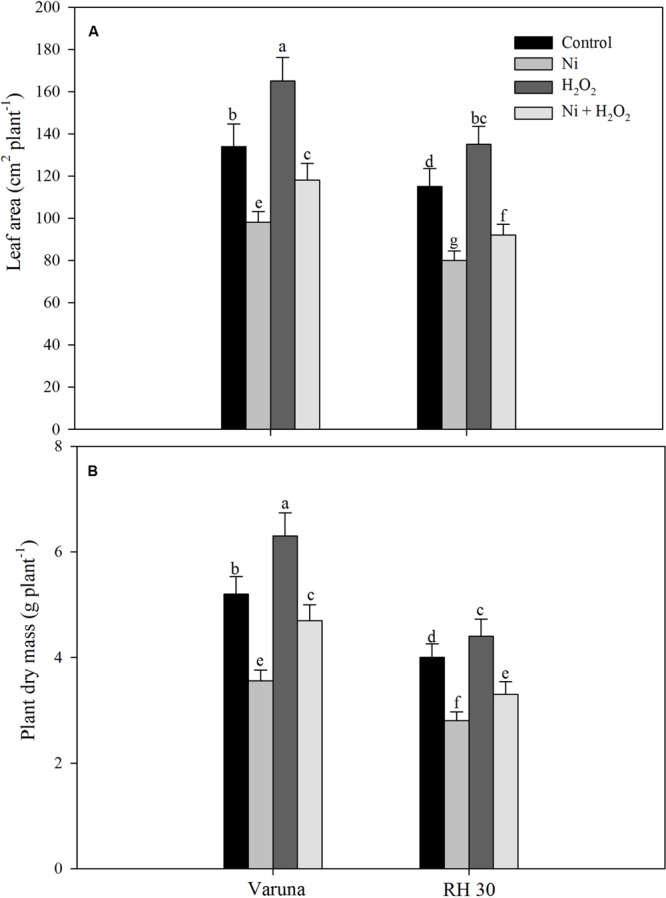
**Leaf area **(A)** and plant dry mass **(B)** of Varuna (high photosynthetic capacity) and RH30 (low photosynthetic capacity) cultivars of mustard (*Brassica juncea* L.) at 30 DAS.** Plants were basally treated with 0 or 50 μM H_2_O_2_ in presence or absence of 200 mg Ni kg^-1^ soil at 15 days after seed germination. Data are presented as treatments mean ± SE (*n* = 4). Data followed by same letter are not significantly different by LSD test at *P* < 0.05.

Leaf area and plant dry mass reduced with Ni treatment in both the cultivars, but more prominently in RH30 compared to control. Application of hydrogen peroxide resulted in reduction in adverse effects of Ni in both the cultivars and increased leaf area by 20.4% in Varuna and by 32.4% in RH30 and plant dry mass by 32.1% in Varuna and by 28.6% in RH30 as compared to Ni-treated plants. Under no stress, application of H_2_O_2_ increased leaf area and plant dry mass by 28.4 and 25.3% in Varuna and by 19.4 and 18.1% in RH30, respectively compared to the Ni treated plants (**Figure 11**).

A comparison of the percent changes in the observed parameters due to H_2_O_2_ in alleviation of Ni stress showed that the effect was more prominent in high photosynthetic cultivar Varuna than the low photosynthetic cultivar RH30 (**Table 3**).

**Table 3:** Percent changes in the parameters studied of Varuna (high photosynthetic capacity) and RH30 (low photosynthetic capacity) cultivars of mustard (*Brassica juncea* L.) at 30 DAS.

	1A		1B	
	**H_2_O_2_ (compared to control)**		**Ni + H_2_O_2_ (compared to Ni)**	
**Parameters**	**Varuna**	**RH30**	**Varuna**	**RH30**

TBARS content	75 ± 3.8*↓	28 ± 1.6↓	33.± 9 1.8*↓	20.5 ± 1.2↓
Electrolyte leakage	50 ± 2.5*↓	41 ± 2.2↓	37.5 ± 2.0*↓	22.4 ± 1.3↓
Ascorbate peroxidase	163.6 ± 8.2*↑	95.4 ± 4.9↑	129.4 ± 6.6*↑	75 ± 3.9↑
Glutathione reductase	69.6 ± 3.5*↑	41.2 ± 2.2↑	69 ± 3.6*↑	50 ± 2.7↑
GSH content	35.9 ± 1.9*↑	14.4 ± 0.9↑	31.7 ± 1.7*↑	17.4 ± 0.9↑
GSSG content	22.0 ± 1.4*↓	30.9 ± 1.7↓	37.4 ± 2.0*↓	32.9 ± 1.8↓
Redox state	74.4 ± 3.8*↑	65.3 ± 3.4↑	111.1 ± 5.8*↑	72.9 ± 3.8↑
ATP-S activity	68.8 ± 3.5*↑	46.2 ± 2.5↑	16.7 ± 0.9↑	13.3 ± 0.8↑
S content	21.3 ± 1.8*↑	9.8 ± 0.6↑	28.6 ± 1.6*↑	9.4 ± 0.6↑
NR activity	15.3 ± 0.9*↑	8.4 ± 0.5↑	17.9 ± 0.9*↑	10.2 ± 0.6↑
N content	22.5 ± 1.4↑	26 ± 1.5↑	24.8 ± 1.4↑	28.3 ± 1.5↑
Photosynthetic-NUE	25.8 ± 1.5*↑	17.0 ± 1.1↑	39.2 ± 2.1↑	28.3 ± 2.2↑
Photosynthetic-SUE	27.6 ± 1.5*↑	18.2 ± 1.0↑	29.2 ± 1.6*↑	21 ± 1.1↑
Net photosynthesis	29.2 ± 1.7*↑	14.1 ± 0.9↑	43.6 ± 2.3*↑	30.9 ± 1.7↑
Stomatal conductance	18.2 ± 1.1*↑	10.6 ± 0.6↑	28.7 ± 1.6*↑	20 ± 1.2↑
Intercellular CO_2_	25 ± 1.4*↑	13.5 ± 0.8↑	30.3 ± 1.8*↑	21.3 ± 1.4↑
Rubisco activity	29.2 ± 1.7*↑	18.9 ± 1.3↑	32.3 ± 1.9*↑	22.7 ± 1.5↑
Chlorophyll content	28.3 ± 1.7*↑	16.7 ± 1.1↑	23.4 ± 1.3*↑	16.3 ± 1.0↑
Actual PSII efficiency	9.8 ± 0.6↑	7 ± 0.4↑	22.4 ± 1.4↑	16 ± 1.0↑
Maximum PSII efficiency	3.2 ± 0.2↑	2.6 ± 0.3↑	5.7 ± 0.4↑	4.3 ± 0.4↑
Intrinsic PSII efficiency	2.5 ± 0.2↑	1.5 ± 0.2↑	8.3 ± 0.5↑	6.7 ± 0.4↑
Electron transport rate	9.9 ± 0.5↑	7.4 ± 0.4↑	22.5 ± 1.3↑	15.9 ± 1.0↑
Photochemical quenching	3.5 ± 0.2↑	2.6 ± 0.2↑	12.2 ± 0.8↑	9.6 ± 0.5↑
Non-photochemical quenching	9.6 ± 0.5↓	12.8 ± 0.8↓	32.3 ± 1.8↓	22.8 ± 1.9↓
Leaf area	23 ± 1.3*↑	17.4 ± 1.1↑	20.4 ± 1.2*↑	15 ± 0.9↑
Plant dry mass	21.2 ± 1.2*↑	10 ± 0.6↑	32 ± 1.7*↑	17.9 ± 1.1↑

Data are presented as treatments mean ± SE (*n* = 4). Data followed by * shows significant difference between the two cultivars by LSD test at *P* < 0.05. Symbols ↑ and ↓ represent increase and decrease, respectively.

## Discussion

Hydrogen peroxide has been known as one of the factors responsible for generating oxidative stress in plant cells (Dat et al., 2000), but the recent studies have shown that it also acts as a signaling molecule participating in various response to abiotic stress tolerance (Gechev and Hille, 2005; Zhang et al., 2011; Zhou et al., 2012). It acts as a signaling molecule at lower concentrations, while provokes the onset of cell death at higher concentrations (Gechev and Hille, 2005). It regulates a number of physiological processes, such as acquisition of resistance, strengthening of cell wall, photosynthesis, and growth of plants (Cheeseman, 2007; Ślesak et al., 2007; Gao et al., 2010; Hossain et al., 2015). The requirement of H_2_O_2_ for better acclimation, improved survival and growth performances was shown for wheat (He et al., 2009) and rice (Hossain and Fujita, 2013) grown under drought stress, for wheat (Wahid et al., 2007; Ashfaque et al., 2014) and rice (Uchida et al., 2002) grown under salt stress, for maize (Wahid et al., 2008) and rice (Uchida et al., 2002) grown under heat stress and mung bean plants under chilling stress (Yu et al., 2003).

Nickel is a micronutrient and plays important roles in plant metabolism. But, recent study of Khan and Khan (2014) have shown that high Ni concentration leads to excess production of ROS and reduction in photosynthesis and growth in mustard plants by reducing the activity of PS II and Rubisco. In the present study, it was found that Ni at 200 mg kg^-1^ soil proved toxic for photosynthesis and growth of both the mustard cultivars, apparently due to high oxidative stress as shown by TBARS content. It was also found that 50 μM H_2_O_2_ proved most effective in increasing photosynthesis and growth of mustard. Earlier reports also demonstrated that low H_2_O_2_ concentration improved salt and heat tolerance of rice plants by enhancing antioxidant system (Uchida et al., 2002) and salt tolerance in wheat by improved nutrient status (Wahid et al., 2007). Hossain and Fujita (2013) found that 50 μM H_2_O_2_ induced defense response in mustard seedlings by activation of methylglyoxal detoxification pathways under drought stress. Thus, our study is consistent with the previous studies showing detoxifying effects of H_2_O_2_ at low dose. Therefore, subsequent study was conducted by taking into account the involvement of high beneficial dose of H_2_O_2_ in modulation of photosynthetic responses in cultivars differing in photosynthetic capacity by monitoring its influence on oxidative stress, N and S-assimilation and photosynthetic-NUE and-SUE.

Nickel treatment increased TBARS content and electrolyte leakage in both the cultivars with greater values in RH30 than Varuna, but exogenously applied hydrogen peroxide lowered TBARS content and electrolyte leakage in both the cultivars with greater response in Varuna. In both the cultivars antioxidant defense system was induced upon exposure to Ni toxicity to avoid damage from generation of excess ROS. Application of H_2_O_2_ enhanced the activity of APX, GR and content of GSH more prominently in Varuna under Ni stress (**Table 3**) which helped in reducing TBARS content and electrolyte leakage. Moreover, more conspicuous increase in the activity of GR by H_2_O_2_ in Varuna indicated higher amount of GSH production in this cultivar to eliminate the products of lipid peroxidation. The activity of GR has been reported to be associated with alteration of the cellular redox status and decisive in determining plant resistance to Ni stress (Khan and Khan, 2014). Also, GR maintains homeostasis of GSH and GSSG crucial for signaling stress response and regulating oxidative stress. Exogenous application of H_2_O_2_ has been shown to promote the expression of stress-response genes and increase Ni stress tolerance. Gao et al. (2010) found that exogenously sourced H_2_O_2_ induced antioxidative enzymes, GR, DHAR, and MDHAR under heat stress condition. The increase in tolerance to abiotic stresses such as heat, chilling, and salts by exogenous application of H_2_O_2_ has been reported in different plants through increase in the activity of antioxidants and reducing peroxidation of membrane lipids (Uchida et al., 2002; de Azevedo Neto et al., 2005; Wahid et al., 2007). Up-regulation of antioxidative defense system by passive absorption of H_2_O_2_ in germinating seeds has been shown to offset oxidative damage leading to improved physiological attributes (Wahid et al., 2007, 2008). The other aspect of increase in GSH content by H_2_O_2_ in both the cultivars under Ni stress was the increase in ATP-S activity. It has been reported that increased GSH content counteracted the Cd-induced oxidative stress with lesser damages to photosynthesis in mustard (Masood et al., 2012; Asgher et al., 2014).

In the present study, application of H_2_O_2_ increased photosynthesis of plants treated with Ni largely because of increase in the activity of Rubisco and PS II. However, the smaller increase in stomatal conductance and intercellular CO_2_ concentration in these plants may not wholly account for the larger increase in photosynthesis, suggesting the involvement of non-stomatal limitations as well for the increase in net photosynthesis. These effects were more pronounced in high photosynthetic capacity cultivar Varuna than the low photosynthetic capacity cultivar RH30. The demand for N and S increased under Ni stress and increased the utilization of N and S more effectively in Varuna than RH30 and proved more beneficial in the alleviation of Ni-induced oxidative stress and protection of photosynthesis under Ni stress condition. The increased N and S assimilation resulting from H_2_O_2_ application accounted for higher NUE and SUE that caused greater photosynthesis through its incorporation into Rubisco more conspicuously in Varuna. The increase in N and S assimilation contributes to chlorophyll biosynthesis, Rubisco activity and in the regulation of photosynthesis (Marschner, 1995). Deprivation of either N or S may cause a significant reduction in the photosynthetic efficiency of plants (Resurreccion et al., 2001; Lunde et al., 2008). The relationship between N content and photosynthesis has been observed in mustard plants (Iqbal et al., 2011), which is also correlated with Rubisco content and S assimilation (Sexton et al., 1998; Iqbal et al., 2012). Higher allocation of N to leaf through the increase in the activity of NR with application of H_2_O_2_ under Ni stress in Varuna increased photosynthesis. It has been suggested that plants with lower photosynthetic-NUE have a lower ability to allocate N to the photosynthetic machinery (Takashima et al., 2004). However, reports on the influence of H_2_O_2_ on photosynthetic-NUE and -SUE in cultivars differing in photosynthetic capacity and grown with Ni in the two cultivars are not known. In the present study, H_2_O_2_ treatment increased net photosynthesis in Varuna greater than RH30 through the greater increase in N and S assimilation and photosynthetic-NUE and -SUE under Ni stress (**Table 3**). Li et al. (2011) observed that exogenous H_2_O_2_ treatment decreased the deleterious effect of salt stress on growth of wheat.

The decrease in chlorophyll content with Ni treatment possibly decreased the absorption of light by the chloroplast and thus indirectly impaired photosynthesis. Photosystem II (PS II) is uniquely vulnerable to the damage by metal stress and chlorophyll fluorescence parameters are a reliable indicator of the intensity of abiotic stress. Chlorophyll fluorescence parameters such as actual efficiency of PS II (ΦPSII), maximum efficiency of PS II (Fv/Fm), and intrinsic efficiency of PS II (Fv’/Fm’) are related to photosynthetic efficiency (Maxwell and Johnson, 2000). Here we found that RH30 was more prone to Ni stress than Varuna as shown by greater decrease in chlorophyll fluorescence parameters (ΦPSII, Fv/Fm, Fv’/Fm’, ETR, and qP) in RH30, and the observed reduction in photosynthesis was mainly attributable to photo-inhibition during the Ni stress. In contrast, NPQ increased under Ni stress with prominent increase in RH30 than Varuna. Ni-induced photo-inhibition and reduced photosynthetic efficiency was reversed by the exogenously sourced H_2_O_2_. The present work demonstrated that application of H_2_O_2_ caused up-regulation of antioxidant system (APX and GR activity and GSH content) and mitigated oxidative damage caused by Ni. The alleviation of oxidative stress by H_2_O_2_ is reflected by significant improvement in early growth characteristics of mustard cultivars most pronounced in high photosynthetic potential cultivar (Varuna) than low photosynthetic potential cultivar (RH30; **Table 3**). The outcome of the study as considering H_2_O_2_ as signaling molecule will help in developing an agricultural friendly technique to overcome the effects of Ni stress on photosynthesis and plant dry mass.

## Conclusion

Conclusively, it may be said that low concentration of H_2_O_2_ induces Ni tolerance by avoiding oxidative stress through increased capacity for N and S assimilation with greater photosynthetic-NUE and -SUE more prominently in Varuna than RH30. The induced activity of ATP-S and antioxidant enzymes (APX and GR) by H_2_O_2_ helped in higher GSH production in Varuna than RH30 and reversed the Ni stress more efficiently. These characteristics of Varuna helped in protecting photosynthesis and maintaining high plant dry mass than RH30. The lower induction of photosynthesis by H_2_O_2_ in RH30 was related to a lesser N and S assimilation and lesser GSH production. The information on the physiological response of mustard in relation to N and S assimilation differing in photosynthetic potential cultivars could be used in understanding the key role of H_2_O_2_ in increasing photosynthesis and growth under Ni stress. Moreover, studies should be focussed to unravel the positive role of H_2_O_2_ in increasing photosynthesis utilizing molecular tools.

## Author Contributions

MK designed the experiment, carried out the analyses and prepared the manuscript. NK supervised the work and was involved in the design of the experiment, preparation, and presentation of the manuscript. AM, TP, and MA carried out the experimental work and searched literature for the work on which this article is based.

## Conflict of Interest Statement

The authors declare that the research was conducted in the absence of any commercial or financial relationships that could be construed as a potential conflict of interest.
